# Active Commuting Behaviors in a Nordic Metropolitan Setting in Relation to Modality, Gender, and Health Recommendations

**DOI:** 10.3390/ijerph121215008

**Published:** 2015-12-09

**Authors:** Erik Stigell, Peter Schantz

**Affiliations:** 1 Research Unit for Movement, Health and Environment, The Swedish School of Sport and Health Sciences, GIH, Stockholm SE-114 86, Sweden; stigell.erik@gmail.com; 2 Unit for Occupational and Environmental Medicine, Department of Public Health and Clinical Medicine, Umeå University, Umeå SE-901 87, Sweden

**Keywords:** walking, bicycling, commuting, distance, duration, velocity, frequency, seasonality, modality

## Abstract

Active commuting between home and place of work or study is often cited as an interesting source of physical activity in a public health perspective. However, knowledge about these behaviors is meager. This was therefore studied in adult active commuters (*n* = 1872) in Greater Stockholm, Sweden, a Nordic metropolitan setting. They received questionnaires and individually adjusted maps to draw their normal commuting route. Three different modality groups were identified in men and women: single-mode cyclists and pedestrians (those who only cycle or walk, respectively) and dual-mode commuters (those who alternately walk or cycle). Some gender differences were observed in trip distances, frequencies, and velocities. A large majority of the commuting trip durations met the minimum health recommendation of at least 10-minute-long activity bouts. The median single-mode pedestrians and dual-mode commuters met or were close to the recommended weekly physical activity levels of at least 150 minutes most of the year, whereas the single-mode cyclists did so only during spring–mid-fall. A high total number of trips per year (range of medians: 231–389) adds to the value in a health perspective. To fully grasp active commuting behaviors in future studies, both walking and cycling should be assessed over different seasons and ideally over the whole year.

## 1. Introduction

Active commuting, *i.e.*, walking and cycling to work or school, is recommended as a source of physical activity by the World Health Organization [1]. It is, in principle, easy to include in daily routines and might therefore be a strategy to overcome the frequently reported “lack-of-time” barrier to physical activity, see, e.g., [2]. Furthermore, walking and cycling do not contribute to such public health problems as climate heating, noise, pollution, and congestion, and pose no severe safety threats to other road users. It is therefore relevant to ask whether active commuting can be the answer to population health, as Professor Roy Shephard did some years ago [3]. He concluded that before answering the question, “we need a more detailed picture of the typical dose of exercise arising from such activity (the typical duration and intensity of bouts, and number of times performed per week)”.

This needs to be studied in different sociodemographic groups, see, e.g., [4], climates, light conditions, topographies, and in the spectrum spanning rural and urban settings on different scales. The reason for the latter need is simply that commuting distances will vary with the scale of a densely built-up area and that this will affect the behaviors as well as the proportions of the populations for which active commuting is a conceivable behavior.

Our study of active commuting behaviors deals with a metropolitan setting in a Nordic country (Greater Stockholm, Sweden) with about 1.9 million inhabitants and distinct variations in temperature as well as daylight over the year. We assumed that within such a widespread built-up area, it was likely that three different groups of active commuters might exist, namely: (1) those who only walk; (2) those who only cycle; and (3) those who alternately walk and cycle. The aim of the study was to explore whether such modal groups existed and to describe their active commuting behaviors (trip distance, duration, frequency, velocity, and red-light stops) over the year. We also analyzed whether or not there were any differences between these variables depending on gender and modal group. It was of specific interest to illuminate the extent to which these behaviors contributed to meeting the minimal level recommendations for moderate physical activity of an at least 10-minute-long physical activity session and a total of 150 min per week for males and females [5,6]. The commuting pattern during a day is often a trip to work in the morning and a trip back home in the evening. By combining these physical activity sessions, there is a potential for achieving both the minimal recommendation of at least 30 min of physical activity per day and the more optimal levels of physical activity of about 60 min per day [5]. The potential of the commuting behaviors for meeting these volumes of physical activity will also be analyzed.

To our knowledge, these dimensions of active commuting behaviors have not been investigated before. A population-based sample would have been ideal for studying these issues. However, given that active commuters constitute a small proportion of the population, this was not feasible. We have, instead, made use of an advertisement-recruited sample.

## 2. Methods and Materials

The present investigation is part of a multidisciplinary study, *i.e.*, Physically Active Commuting in Greater Stockholm (PACS) by the Research Unit for Movement, Health and Environment at the Swedish School of Sport and Health Sciences, GIH, in Stockholm, Sweden [7]. By the term active commuting, we mean trips by foot or bicycle the whole way between home and place of work or study.

### 2.1. Study Area

The study area is the County of Stockholm, Sweden, with the exception of the municipality of Norrtälje. This metropolitan area, with about 1.9 million inhabitants, consists of inner urban, suburban, and rural areas. The latter two areas will be referred to in the following as “suburban areas”. The geographic distinction between the inner urban and suburban areas is given in Wahlgren *et al.*, 2010 [8]. In 2004, the year when the main part of the study was undertaken, about 285,000 people lived in the inner urban area [9]. It also had a high density of workplaces and employees and is socio-economically linked with the surrounding territory by, e.g., commuting. A detailed environmental description of the study area has been given in Wahlgren and Schantz [10]. Here we describe the topography, urban form, and residential density, as well as the weather and daylight variability over the year. The reason for this is that these are variables that can affect active commuting behaviors.

The natural landscape in the region is basically rather flat. The road system includes, however, rather gentle slopes of infrequent moraine hills, normally not accounting for more than about 10–15 m of elevation. The inner urban area is a predominantly built-up area, with blocks in a grid-like pattern. In 2005, the residential density was approximately 13,000 residents per square km in the inner urban area [11]. The suburban and rural areas contained a mixture of residential areas, smaller industrial areas and managed forests, as well as agricultural land. The streets are not normally laid out in a grid-like pattern. Instead, the main roads often follow old road networks formed during the agricultural period of the landscape. As a representation of the residential density of the suburban parts of the study area, we have chosen the southern and western suburbs of the Municipality of Stockholm; in 2005 they accounted for approximately 3500 and 2900 residents per square km, respectively [11]. The weather and daylight conditions during four months representing distinctly different parts of the year prior to the actual survey month of September 2004, and for a 30 year long period are given in Table 1.

**Table 1:** Temperature, precipitation, and daylight during four months in Stockholm, Sweden (mean values).

Months	October	December	March	June
Temperature, °C ^**a**/**b**^	5/8	1/−1	1/0	14/16
Precipitation, >1 mm/24 h ^**a**/**b**^	3/9	12/10	6/7	9/7
Sunrise, time ^**c**,**d**^	07.41	08.47	05.50	03.34
Sunset, time ^**c**,**d**^	17.23	14.45	18.01	22.05

**^**a**^** Mean values for four months in 2003–2004 prior to the participants’ descriptions of their active commuting behaviors in September 2004, Source: Eastern Sweden´s Air Quality Management Association [12]; **^**b**^** Mean values for 1961–1990, Source: Hong Kong Observatory [13]; **^c^** Source: Air Navigation Services of Sweden [14]; **^d^** Times for the 21st day in the month.

### 2.2. Participants

Given that bicycle commuters constitute only a small proportion of the population, it was not feasible to make a population-based study. Instead, we had to elaborate on how to capture the phenomenon of active commuting in a metropolitan setting. Recruitment in the street was considered to be one option. However, a widespread geographic coverage is difficult to achieve. Instead, we decided to recruit primarily via advertisements in newspapers, and then we compared the results obtained in that fashion with those from a street-recruited sample of bicyclists (see Discussion).

The study is based on the responses of 2148 responders to advertisements in two morning newspapers (Svenska Dagbladet and Dagens Nyheter) in May 2004. They sent in a response coupon from the advertisement with written addresses to their home and place of work or study. The inclusion criteria were: participants should be of a minimum age of 20 years, live in the County of Stockholm, except for the municipality of Norrtälje, and walk or cycle the whole way to their place of work or study at least once a year. There was no maximum age limitation for participation. The information to the commuters emphasized that people with very short commuting distances were also welcome to participate.

The Ethics Committee of the Karolinska Institute approved the study, and the participants gave their informed consent.

### 2.3. Questionnaires, Administration, Response Rates, and Inclusion Criteria

The participants responded to two different questionnaires created for the study, Physically Active Commuting in Greater Stockholm (PACS). The questionnaires were pre-tested on a small convenience sample of academic staff members as part of the process of developing them. They are referred to as PACS Q1 and PACS Q2. PACS Q1 was sent out in September 2004, and PACS Q2 in May and June 2005.

PACS Q1 is self-administered, in Swedish, and contains 35 items, of which we only used a few in this study, namely: gender, height, weight, year of birth, employment status, average frequency of commuting trips per week, by month, over the year, active commuting durations to work, whether a single or double active commute trip per day was the predominant behavior, and number of stops at red lights. For the questions related to active commuting behaviors, there were separate response alternatives for walking and cycling.

Other data were taken from responses to PACS Q2, which is self-administered, in Swedish, and contains 70 items (including the Active Commuting Route Environment Scale (ACRES), described in detail in [8]). Here we used only a few items from PACS Q2, namely: level of education, income, and ethnic background, access to a car, driver's license, physical health and mental health, and the times for departure from home and place of work or study.

Out of the original 2148 responders to the advertisement, 133 did not return the PACS Q1, giving a response rate of 93.8%. Twenty-one of the responders did not meet the inclusion criteria. At the end of PACS Q1, we asked if they were willing to participate in the second part of the study, which was going to be based on PACS Q2. Based on their responses in this respect, 1994 participants received and returned PACS Q2, giving a response rate of 100% in this second step of the survey. Out of these respondents, we excluded 122 with missing values on commuting time or trip frequency, or with extreme commuting frequencies exceeding 20 trips per week in any month, which led to a total of 1872 participants in this study.

### 2.4. Maps and Route Distance Measurements

The initial respondents sent in response coupons with their written home and place of work or study addresses. Based on this, we prepared an individually adjusted map for each participant, with the aim that they should draw their own active commuting routes on it (see below). The maps were sent out in September 2004 together with PACS Q1. It was a black and white copy of maps from the regular Stockholm telephone directory (scale 1:25,000). In 25 cases the standard national outdoor map (scale 1:50,000) was used since the relevant area was not found on the maps in the Stockholm telephone directory. On the maps, the participants marked their most usual commuting routes with different markings for bicycle and pedestrian routes, as well as for the trip to and from their place of work or study, respectively. Furthermore, they marked their homes on the maps with the capital letter B and their places of work or study with a small box. The method used in this respect has been described in detail in Schantz and Stigell (2009) [15]. We used the maps to measure route distances (see below). Participants with two or more places of work or study were asked to pick the one they spent the most time at and, in the case of equal time spent, to choose one of them. They were then asked to walk or cycle their route once, noting their route choice and the street names, before filling in the routes on the map. Finally, the respondents were asked to carefully mark their routes in case their routes included places outside of the printed street grid network, such as parkways or tunnels.

The technical device used for the distance measurements was a digital curvimetric instrument (Run Mate Club, CST/Berger, Watseka, IL, USA) applied within the context of a criterion method with high validity and reproducibility [15]. The distances of the map-drawn commuting routes from home to places of work or study were measured twice by a technical assistant, and if the two measurements differed, a third measurement was undertaken. This was done in 151 cases with differences between the first two values being greater than 5.8 ± 3.3 mm (mean ± SD). We then used the mean value of the two closest values of the three.

### 2.5. Categorization into Mode Groups

The possibility of different behaviors with different transport modes motivated us to divide the participants into three mode groups, based on how they reported their commuting mode in the PACS Q1 questionnaire, *i.e.*, either only cycling (single mode cyclists) or only walking (single mode pedestrians), and, for those who used both modes alternately, *i.e.*, walked the whole way sometimes, and cycled it at other times, we formed a separate category: the dual-mode group. We based the mode categorization on answers to a question about “commuting time”, divided into separate response alternatives for walking and cycling, and verified the categorization from the answers to “commuting frequency”, “stated distance”, and route markings on maps. Finally, 139 participants with somewhat uncertain categorizations were mailed a follow-up question.

### 2.6. Categorization of Origins and Destinations in the Different Mode Groups

For interpretation of the results, it was considered that it may be useful to distinguish between where within the study area the different modality groups were executing there commuting trips. One reason for this was the noted distinct differences in perceived and rated route environmental variables, such as mixed traffic congestion, flow and speed of motor vehicles, and number of bicycle paths, between the inner urban and suburban areas [10], which, in principle, can affect such variables as velocity of cycling. Therefore, based on the postal area codes in the addresses to the participants’ homes and places of work or study, the origins and destinations of the trips in the morning were categorized for each modality group in relation to the inner urban and suburban areas. For the spatial delimitations between these areas, see [8].

### 2.7. Frequency of Trips, Commuting Durations, and Red Lights

On the questionnaire, the participants also marked their normal commuting frequency on an array of pre-specified numbers representing the mean of commuting trips per week for each month. The response options were whole numbers from 0 to 14, a free response option and an option “Less than one”, to which we assigned the value 0.5. In cases where the participant had some responses and some non-responses in the array, we interpreted the non-responses as zeroes. This did not change the general pattern of zero commuting times in the samples since most of these non-responses were in the holiday month of July and in the winter. The numbers of commuting trips per year were calculated by adding up the weekly frequencies for each month, weighted in relation to the number of days in the different months. The mean weekly frequency was calculated by adding up the weekly frequencies of all months and dividing the total by 12.

Participants were asked to record the times for their commuting trips on a normal day and when no other errands were undertaken. They wrote down their commuting time to their place of work or study in hours and minutes separately for walking and cycling. In the inspection of time data, we found that 75% of the reported times were multiples of five or ten minutes, probably mostly due to rounding off. The total weekly commuting time by month was calculated by multiplying the stated commuting duration with the stated weekly mean trip frequency by month.

The participants were asked to count the number of stops at red lights on a normal day, prior to reporting it.

### 2.8. Characteristics of the Participants

The characteristics of the participants are described in Table 2. Sixty-eight percent were women and 58% were cyclists, 15% pedestrians and 27% dual-mode commuters. Eighty-seven percent of the participants reported that they depart from home sometime between 6:00 a.m. and 9:00 a.m. and 89% that they start their return trip from work between 3:00 p.m. and 7:00 p.m. These times were reported for two weeks in May and June when the participants responded to PACS Q2, but they are probably also valid for the rest of the working days of the year.

**Table 2:** Participant characteristics.

	Men			Women		
	Walk	Cycle	Dual Mode	Walk	Cycle	Dual Mode
Range of Number of Responses: *	55–63	417–464	75–83	181–214	583–660	331–388
Age in years, mean ± SD	49 ± 10	47 ± 11	47 ± 12	49 ± 10	46 ± 11	47 ± 11
Weight in kg, mean ± SD	80 ± 11	79 ± 10	80 ± 10	65 ± 10	65 ± 8	65 ± 9
Height in cm, mean ± SD	180 ± 7	181 ± 6	181 ± 7	167 ± 5	168 ± 6	168 ± 6
Body mass index, kg/m^2^, mean ± SD	24 ± 3	24 ± 3	24 ± 3	23 ± 3	23 ± 3	23 ± 3
Gainful employment, %	95	94	92	97	95	95
Educated at university level, %	75	70	76	72	76	71
Income above 25,000 SEK ^**‡**^ a month, %	56	66	64	51	55	53
Participant and both parents born in Sweden, %	86	82	80	86	83	81
Having a driver’s license, %	94	94	92	92	92	90
Usually access to a car, %	65	79	71	57	73	64
Overall physical health good or very good, %	83	81	91	87	80	79
Overall mental health good or very good, %	81	86	83	88	80	80
Live in inner urban area, %	57	20	48	65	24	42
Work/study in inner urban area, %	70	54	58	68	45	47

* = This range is due to the fact that respondents sometimes did not answer particular questions. **^‡^** SEK = the currency of Sweden, 2005: 1 Euro ≈ 10 SEK; 1 USD ≈ 8 SEK.

In Table 3, we display the mode choice groups’ different geographical origin and destination patterns and, in Table 4, the relative distribution of the number of bicycle gears for the mode choice groups and gender is indicated.

**Table 3:** Origin and destination scheme of the participants.

Group	Origin	Destination	
		Inner Urban	Suburban
Pedestrians (*n* = 277)	Inner urban, %	58	5
	Suburban, %	10	27
Cyclists (*n* = 1124)	Inner urban, %	12	10
	Suburban, %	36	42
Dual Mode (*n* = 471)	Inner urban, %	37	6
	Suburban, %	12	45

**Table 4:** Description of types of bicycles used.

Type of Bicycle	Single Mode Cyclists		Dual Mode Cyclists	
	Men *(n* = 438)	Women (*n* = 627)	Men *(n* = 80)	Women (*n* = 367)
No gears, %	3	7	11	11
2–4 gears, %	9	27	21	39
5 gears or more, %	88	66	68	50

### 2.9. Statistical Analyses

Values are presented as medians together with the first and third quartiles and means ± 1 standard deviation (SD). The possibility of significant gender differences in the variables, *i.e.*, distance, duration, velocity, red-light stops, and numbers of commuting days per year, weekly trip frequency by month, and total weekly commuting time per week by month, as well as the mean value per year, were tested with the Mann–Whitney test for each mode group. The possibility of significant differences between dual and single-mode commuters in the same variables was tested using the Mann–Whitney test for each gender group separately for walking and cycling. In the Mann–Whitney tests, we applied a Bonferroni correction that changed the significance level to *p* < 0.025, which corresponds to the chosen significance level of 0.05 divided by the number of comparisons made, *i.e.*, two, and, in the text, this will be referred to as statistically significant. In all statistical analyses, we used IBM SPSS Statistics 17.0 (SPSS Inc., Chicago, IL, USA).

## 3. Results

Three different categories of active commuters were noted. These categories are single mode pedestrians, single mode cyclists and dual mode commuters who alternately walk and cycle to work.

**Table 5:** Participants’ commuting distance, duration, velocity, commuting trips per year and red-light stops, median values (first–third quartile) and mean values ± 1 SD. In addition, the proportions of one-way commuting-trip durations of 10, 15, and 30 min or longer, respectively, within the modal groups are indicated. The extent to which two active commuting trips per day constitute the commuters’ normal behaviour is also indicated as proportions within each modal group.

Behavioral Variables	Walking				Cycling			
	Single Mode		Dual Mode		Single Mode		Dual Mode	
	Men	Women	Men	Women	Men	Women	Men	Women
	*n* = 63	*n* = 214	*n* = 83	*n* = 388	*n* = 464	*n* = 660	*n* = 83	*n* = 388
**Distance** (km)								
Median (Q1-Q3)	2.3 (1.7–3.9)	2.3 (1.4–3.4) ^**a**^	2.7 (2.0–4.9)	2.9 (1.9–4.0)	9.0 (5.8–13.8) ^**ab**^	6.7 (4.4–9.8) ^**a**^	2.9 (2.0–4.9)	3.0 (2.0–4.2)
Mean ± 1SD	3.0 ± 2.0	2.5 ± 1.3	3.6 ± 2.5	3.2 ± 1.9	10.6 ± 6.6	7.7 ± 4.5	3.7 ± 2.5	3.3 ± 1.9
**Duration** (minutes)								
Median (Q1-Q3)	25 (20–45)	26 (18–35) ^**a**^	35 (20–50)	35 (25–45)	30 (20–40) ^**a**^	27 (20–40) ^**a**^	14 (9–20)	15 (10–20)
Mean ± 1SD	32.8 ± 19.6	28.8 ± 14.3	38.1 ± 23.9	36.2 ± 19.2	32.8 ± 16.9	30.1 ± 14.7	15.3 ± 8.9	15.3 ± 7.1
**One-way trip durations**								
≥10 min, %	93	97	96	98	98	97	74	84
≥15 min, %	90	89	91	92	91	91	46	55
≥30 min, %	46	47	58	64	53	49	7	5
**Velocity** (km/h)								
Median (Q1-Q3)	5.4 (4.8–5.9) ^**b**^	5.2 (4.6–5.6)	5.4 (5.0–6.0)	5.2 (4.7–5.9)	18.6 (15.7–21.8) ^**ab**^	14.9 (12.6–17.2) ^**a**^	14.3 (11.9–16.9) ^**b**^	12.8 (10.7–14.9)
Mean ± 1SD	5.4 ± 0.8	5.1 ± 0.9	5.6 ± 1.0	5.3 ± 1.0	18.7 ± 4.4	14.9 ± 3.4	14.5 ± 3.8	13.0 ± 3.5
**Trips per day**								
Normally two, %	82	76	60	60	97	96	100	96
**Trips per year**								
Median (Q1-Q3)	376 (217–459) ^**a**^	389 (266–459) ^**a**^	60 (26–172)	86 (30–172)	262 (165–386) ^**b**^	231 (148–346)	277 (190–364)	248 (176–344)
Mean ± 1SD	341 ± 142	358 ± 155	117 ± 133	111 ± 98	275 ± 136	250 ± 140	277 ± 140	256 ± 117
**Red-light stops**								
Median (Q1-Q3)	2 (1–3)	2 (1–4) ^**a**^	1 (0–3)	1 (0–3)	3 (1–5) ^**a**^	3 (1–5) ^**a**^	2 (1–3)	2 (0–3)
Mean ± 1SD	2.0 ± 2.0	2.7 ± 2.7	2.0 ± 2.8	1.8 ± 2.1	3.6 ± 3.1	3.5 ± 3.2	2.4 ± 2.4	2.1 ± 2.2

**^**a**^** = Significant mode group difference within a walking and cycling gender group, respectively; **^**b**^** = Significant gender difference within a mode group. The proportion values have not been subjected to statistical between-group or gender comparisons.

### 3.1. Distance, Duration, Velocity, Trips per Year and Red-Light Stops

Data on trip distance, duration, velocity, trips per year and red-light stops for the different modality groups and gender are given in Table 5. As could be expected, distances and velocities varied greatly between the groups. In contrast, the median trip durations for the single mode cyclists and pedestrians were rather similar, ranging between 25 and 30 min, and was 35 min within the dual mode group when they walked. In most modal groups, nearly all participants had single-commute durations of 10 min or more. About 90% in most groups had a single-trip duration of 15 min or more, and about 50% had a single-trip duration of 30 min or more. The only exception was the dual-mode groups when cycling. The most frequent behavior of all modality groups was to make an active commute trip both to their place of work and home on the same day. High median frequencies of trips over the year were noted for the single-mode pedestrians (376–389). Lower levels were noted in the cycling behaviors described (231–277). On top of these, however, the dual-mode commuters added 60–86 walking trips per year. Larger gender differences (>15%) were noted in single-mode cycling velocities and distances, with velocities and distances for males being 26% and 34% higher, respectively. For further details on modality and gender differences, see Table 5.

### 3.2. Trip Frequency per Week Over the Year

**Figure 1:**
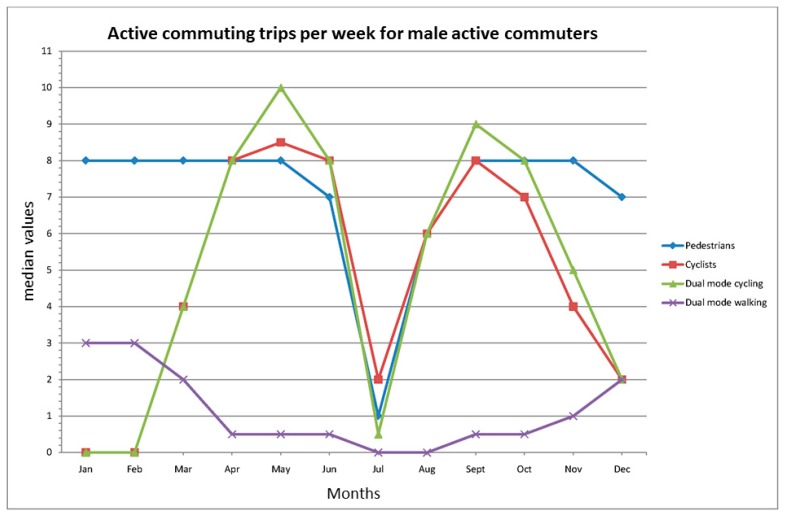
Active commuting frequency of trips per week for male active commuters in different months, median values. The total frequency of walking and cycling trips per week in the dual-mode group is given in Table 6.

**Figure 2:**
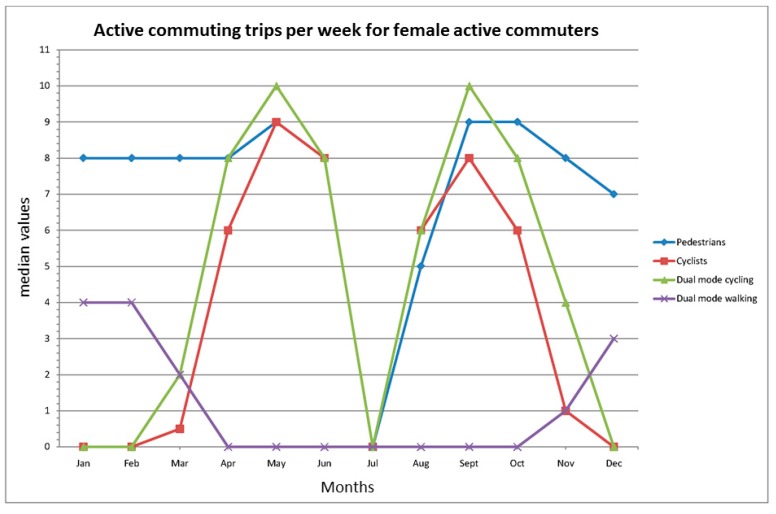
Active commuting frequency of trips per week for female active commuters in different months, median values. The total frequency of walking and cycling trips per week in the dual mode group is given in Table 6.

The trip frequencies per week over the year are illustrated in Figure 1 and Figure 2 and further described in Table 6. High median frequencies of walking trips were noted in both male and female single-mode pedestrians, except for during the vacation month of July, whereas both the single and dual-mode cycling behaviors are predominantly a spring to mid-fall phenomenon. It is notable that when the cycling trip frequency is diminished in the dual-mode groups, they substitute for it with walking trips, but to a lesser extent than the single-mode pedestrians. During the period November–March, male cyclists in both the single and dual-mode groups have low, but higher trip frequencies than females. For further details on modality and gender differences, see Table 6.

### 3.3. Total Time per Week Over the Year

**Figure 3:**
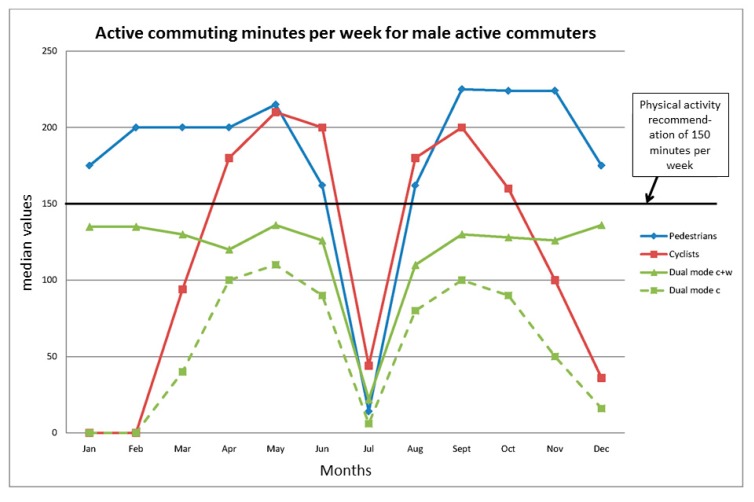
Active commuting time per week for male active commuters in different months, median values. “Dual mode c + w” stands for the combined durations of walking and cycling, whereas “Dual mode c” stands for the levels attained with only cycling. For more details, see Table 7.

**Figure 4:**
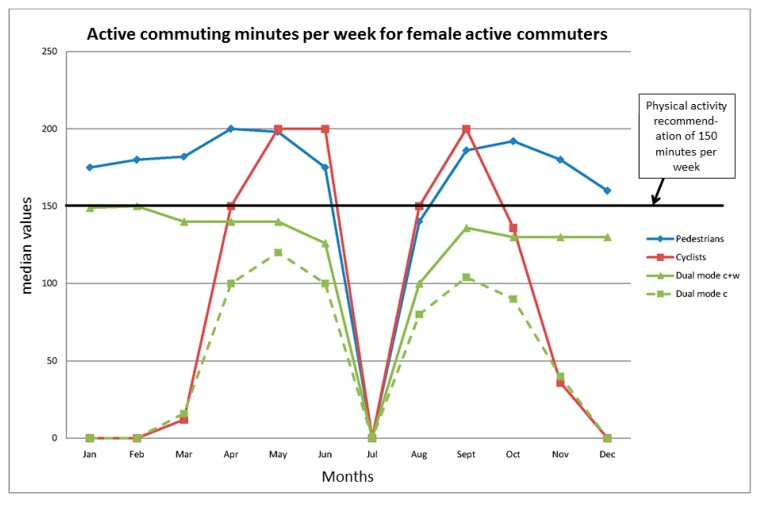
Active commuting time per week for female active commuters in different months, median values. See also Table 7. For an explanation of “Dual mode c + w” and “Dual mode c”, see legend to Figure 3.

Combining trip frequency with duration provides data on the total active commuting time per week for each month over the year within each modality (Figure 3 and Figure 4, Table 7). Figure 3 and Figure 4 also depict the minimal levels of health-enhancing physical activity for 150 min per week. The median time for both male and female pedestrians indicates that they meet these levels in a stable fashion, the only exception being during the predominant vacation month of July. The same is true for the cyclists when their season peaks during about six months (April–June and August–October). The cycling behavior of the dual-mode cyclists mirrors that of the single-mode cyclists with regard to seasonality, but they only come up to about 100 min of cycling per week during that period. However, on combining the cycling with their walking, they reach stable physical activity levels in the order of about 130–140 min per week over the year. For details on modality and gender differences, see Table 7.

**Table 6:** Participants’ number of commuting trips per week over the year and averaged for the the whole year, median values (first–third quartile) and mean values ± 1 SD.

Modal Group		January	February	March	April	May	June	July	August	September	October	November	December	Year
**Walking**														
Single-mode	Men	8 (3–10) ^**a**^	8 (4–10) ^**a**^	8 (4–10) ^**a**^	8 (5–10) ^**a**^	8 (5–10) ^**a**^	7 (4–9) ^**a**^	1 (0–5) ^**a**^	6 (3–9) ^**a**^	8 (5–10) ^**a**^	8 (5–10) ^**a**^	8 (5–10) ^**a**^	7 (3–10) ^**a**^	7 (4–9) ^**a**^
	*n* = 63	6.6 ± 3.6	6.9 ± 3.6	7.2 ± 3.5	7.1 ± 3.3	7.5 ± 3.0	6.3 ± 3.3	3.0 ± 3.6	5.7 ± 3.6	7.5 ± 3.2	7.3 ± 3.3	7.2 ± 3.5	6.5 ± 3.4	6.6 ± 2.7
	Women	8 (5–10) ^**a**^	8 (5–10) ^**a**^	8.5 (6–10) ^**a**^	8 (6–10) ^**a**^	9 (6–10) ^**a**^	8 (5–10) ^**a**^	0 (0–5) ^**a**^	5 (3–9) ^**a**^	9 (5–10) ^**a**^	9 (5–10) ^**a**^	8 (5–10) ^**a**^	7 (5–10) ^**a**^	8 (5–9) ^**a**^
	*n* = 214	7.1 ± 3.6	7.5 ± 3.6	7.7 ± 3.4	7.7 ± 3.3	7.9 ± 3.2	7.1 ± 3.6	2.1 ± 3.3	5.8 ± 3.7	7.8 ± 3.3	7.7 ± 3.6	7.5 ± 3.6	6.8 ± 3.6	6.9 ± 3.0
Dual-mode	Men	3 (0.5–8)	3 (0.5–8)	2 (0.5–5)	0.5 (0–2)	0.5 (0–1) ^**b**^	0.5 (0–1) ^**b**^	0 (0–0.5) ^**b**^	0 (0–1) ^**b**^	0.5 (0–1) ^**b**^	0.5 (0–2)	1 (0–4)	2 (0.5–6)	1 (0.5–3)
	*n* = 83	4.1 ± 3.9	4.2 ± 4.0	3.1 ± 3.5	1.6 ± 2.9	1.5 ± 2.9	1.4 ± 2.9	0.5 ± 1.4	1.2 ± 2.5	1.6 ± 2.9	1.9 ± 3.1	2.6 ± 3.2	3.4 ± 3.6	2.3 ± 2.6
	Women	4 (1–8)	4 (1–9)	2 (0–6)	0 (0–2)	0 (0–0.5)	0 (0–0.5)	0 (0–0)	0 (0–0.5)	0 (0–0.5)	0 (0–2)	1 (0–5)	3 (0.5–8)	2 (0.6–3)
	*n* = 388	4.7 ± 3.9	4.7 ± 4.0	3.4 ± 3.8	1.3 ± 2.3	0.8 ± 1.7	0.7 ± 1.6	0.3 ± 1.1	0.7 ± 1.8	0.9 ± 2.0	1.5 ± 2.7	2.8 ± 3.5	4.0 ± 3.8	2.1 ± 1.9
**Cycling**														
Single-mode	Men	0 (0–6) ^**b**^	0 (0–7) ^**b**^	4 (0–8) ^**b**^	8 (4–10)	8.5 (6–10)	8 (5–10)	2 (0–5) ^**b**^	6 (4–10)	8 (5–10)	7 (3–10)	4 (0–9) ^**b**^	2 (0–6) ^**b**^	5 (3–7) ^**ab**^
	*n* = 464	3.0 ± 3.9	3.2 ± 4.0	4.3 ± 4.1	6.6 ± 3.5	7.8 ± 3.1	7.4 ± 3.0	3.1 ± 3.6	6.5 ± 3.3	7.4 ± 3.2	6.2 ± 3.8	4.6 ± 4.2	3.3 ± 3.7	5.3 ± 2.6
	Women	0 (0–3)	0 (0–4)	0.5 (0–8)	6 (3–10) ^**a**^	9 (6–10)	8 (5–10)	0 (0–5)	6 (4–10)	8 (5–10)	6 (1–10) ^**a**^	1 (0–8)	0 (0–5)	4 (3–7) ^**a**^
	*n* = 660	2.1 ± 3.7	2.2 ± 3.8	3.4 ± 4.2	6.2 ± 3.9	7.8 ± 3.2	7.4 ± 3.3	2.5 ± 3.4	6.3 ± 3.4	7.4 ± 3.5	5.7 ± 4.2	3.8 ± 4.4	2.5 ± 3.9	4.8 ± 2.7
Dual-mode	Men	0 (0–4) ^**b**^	0 (0–4) ^**b**^	4 (0.5–8) ^**b**^	8 (4–10)	10 (6–10)	8 (5–10)	0.5 (0–5)	6 (4–10)	9 (6–10)	8 (4–10)	5 (2–9) ^**b**^	2 (0–6) ^**b**^	5 (4–7)
	*n* = 83	2.5 ± 3.7	2.5 ± 3.8	4.6 ± 3.9	7.3 ± 3.6	8.0 ± 3.3	7.1 ± 3.1	2.7 ± 3.4	6.0 ± 3.4	7.7 ± 3.4	6.9 ± 3.7	5.3 ± 4.1	3.3 ± 4.0	5.3 ± 2.7
	Women	0 (0–0.5)	0 (0–0.5)	2 (0–7)	8 (4–10)	10 (6–10)	8 (5–10)	0 (0–5)	6 (4–10)	10 (6–10)	8 (4–10)	4 (0–8)	0 (0–4)	5 (3–7)
	*n* = 388	1.5 ± 2.9	1.5 ± 3.0	3.4 ± 3.9	7.0 ± 3.6	8.2 ± 2.9	7.7 ± 3.2	2.8 ± 3.6	6.4 ± 3.4	7.8 ± 3.3	6.6 ± 3.9	4.1 ± 4.2	2.0 ± 3.2	4.9 ± 2.2
**Walking + Cycling**														
Dual-mode	Men	7 (3–10)	8 (3–10)	8 (4–10)	10 (8–10)	10 (8–10)	10 (6–10)	2 (0–5)	8 (5–10)	10 (8–10)	10 (7–10)	8 (6–10)	7 (4–10)	8 (6–9)
	*n* = 83	6.6 ± 4.4	6.7 ± 4.4	7.7 ± 4.1	8.9 ± 3.5	9.5 ± 3.1	8.5 ± 3.3	3.2 ± 4.0	7.2 ± 3.8	9.3 ± 3.5	8.8 ± 3.6	7.9 ± 4.2	6.7 ± 4.5	7.6 ± 3.1
	Women	7 (2–10)	8 (2–10)	8 (4–10)	10 (6–10)	10 (8–10)	10 (6–10)	0 (0–6)	8 (5–10)	10 (8–10)	10 (6–10)	8 (4–10)	7 (2–10)	8 (5–9)
	*n* = 388	6.2 ± 4.1	6.2 ± 4.0	6.8 ± 3.7	8.3 ± 3.0	9.0 ± 2.6	8.4 ± 3.1	3.1 ± 3.9	7.2 ± 3.6	8.7 ± 3.0	8.0 ± 3.6	6.9 ± 3.9	6.0 ± 3.9	7.1 ± 2.5

**^**a**^** = Significant mode group difference within a walking and cycling gender group, respectively; **^**b**^** = Significant gender difference within a mode group. The total number of walking and cycling trips in the dual-mode groups have not been compared statistically with the frequency of the single-mode groups of each gender.

**Table 7:** Participants’ commuting time in minutes per week over the year and averaged for the whole year, median values (first–third quartile) and mean values ± 1 SD.

Modal group		January	February	March	April	May	June	July	August	September	October	November	December	Year
**Walking**														
Single-mode	Men	175 (90–250) ^**a**^	200 (90–250) ^**a**^	200 (110–250) ^**a**^	200 (120–270) ^**a**^	215 (120–270) ^**a**^	162 (90–240) ^**a**^	14 (0–165) ^**a**^	162 (45–235) ^**a**^	225 (120–270) ^**a**^	224 (120–270) ^**a**^	224 (119–270) ^**a**^	175 (102–250) ^**a**^	190 (119–248) ^**a**^
	*n* = 63	182 ± 114	189 ± 112	199 ± 112	200 ± 108	211 ± 107	171 ± 113	82 ± 104	162 ± 130	217 ± 125	214 ± 128	207 ± 129	185 ± 120	185 ± 90
	Women	175 (100–244) ^**a**^	180 (120–250) ^**a**^	182 (120–266) ^**a**^	200 (124–250) ^**a**^	198 (130–266) ^**a**^	175 (103–244) ^**a**^	0 (0–100) ^**a**^	140 (75–200) ^**a**^	186 (120–254) ^**a**^	192 (120–250) ^**a**^	180 (120–250) ^**a**^	160 (100–225) ^**a**^	165 (111–230) ^**a**^
	*n* = 214	185 ± 125	195 ± 127	201 ± 125	203 ± 119	210 ± 122	187 ± 126	58 ± 106	155 ± 125	205 ± 124	202 ± 129	194 ± 128	175 ± 125	181 ± 110
Dual-mode	Men	80 (17–174)	81 (17–174)	48 (7–120)	18 (0–60)	10 (0–48) ^**b**^	5 (0–43) ^**b**^	0 (0–12) ^**b**^	0 (0–35) ^**b**^	10 (0–48) ^**b**^	20 (0–70)	30 (0–100)	60 (7–140)	42 (18-79)
	*n* = 83	112 ± 115	112 ± 116	89 ± 113	41 ± 61	41 ± 73	36 ± 71	13 ± 31	31 ± 55	41 ± 68	51 ± 76	71 ± 90	93 ± 105	61 ± 59
	Women	110 (35–210)	116 (35–210)	64 (0–160)	0 (0–60)	0 (0–20)	0 (0–18)	0 (0–0)	0 (0–20)	0 (0–24)	0 (0–70)	38 (0–140)	85 (10–200)	50 (22–92)
	*n* = 388	137 ± 123	139 ± 125	97 ± 108	42 ± 70	27 ± 64	23 ± 53	11 ± 39	25 ± 79	31 ± 84	49 ± 109	82 ± 117	116 ± 120	65 ± 61
**Cycling**														
Single-mode	Men	0 (0–120) ^**b**^	0 (0–139) ^**b**^	94 (0–180) ^**ab**^	180 (100–250) ^**ab**^	210 (150–300) ^**a**^	200 (135–300) ^**ab**^	44 (0–168) ^**ab**^	180 (100–270) ^**a**^	200 (125–300) ^**a**^	160 (80–250) ^**ab**^	100 (0–200) ^**b**^	36 (0–144) ^**b**^	133 (89–201) ^**ab**^
	*n* = 464	74 ± 105	79 ± 111	115 ± 123	192 ± 132	237 ± 136	230 ± 135	99 ± 131	199 ± 136	220 ± 136	178 ± 136	123 ± 126	84 ± 107	153 ± 89
	Women	0 (0–60)	0 (0–63)	12 (0–140)	150 (73–240) ^**a**^	200 (120–280) ^**a**^	200 (120–280) ^**a**^	0 (0–125)	150 (90–250) ^**a**^	200 (110–270) ^**a**^	136 (33–200) ^**a**^	36 (0–150)	0 (0–87)	112 (75–165) ^**a**^
	*n* = 660	41 ± 80	45 ± 85	77 ± 105	168 ± 135	222 ± 141	211 ± 145	74 ± 113	181 ± 131	205 ± 143	146 ± 129	89 ± 116	54 ± 93	126 ± 80
Dual-mode	Men	0 (0–48) ^**b**^	0 (0–60) ^**b**^	40 (6–90) ^**b**^	100 (48–144)	110 (54–152)	90 (48–150)	6 (0–70)	80 (36–120)	100 (54–150)	90 (48–120)	50 (20–108) ^**b**^	16 (0–80) ^**b**^	64 (36–104)
	*n* = 83	42 ± 77	44 ± 88	72 ± 97	112 ± 94	123 ± 95	110 ± 86	45 ± 74	90 ± 72	119 ± 94	105 ± 93	81 ± 96	53 ± 88	83 ± 76
	Women	0 (0–10)	0 (0–10)	16 (0–84)	100 (50–150)	120 (72–158)	100 (65–150)	0 (0–80)	80 (50–125)	104 (64–150)	90 (40–150)	40 (0–100)	0 (0–49)	65 (40–98)
	*n* = 388	22 ± 49	23 ± 50	52 ± 69	104 ± 72	123 ± 69	116 ± 70	47 ± 70	93 ± 64	116 ± 72	97 ± 75	62 ± 75	30 ± 52	74 ± 48
**Walking + Cycling**														
Dual-mode	Men	135 (70–240)	135 (70–248)	130 (71–250)	120 (88–200)	136 (94–204)	126 (70–190)	22 (0–94)	110 (55–160)	130 (88–200)	128 (88–192)	126 (80–210)	136 (60–210)	118 (83–198)
	*n* = 83	154 ± 112	157 ± 119	160 ± 122	153 ± 102	164 ± 105	145 ± 100	58 ± 83	121 ± 88	159 ± 108	157 ± 103	151 ± 107	146 ± 113	144 ± 88
	Women	149 (66–234)	150 (68–240)	140 (80–200)	140 (88–200)	140 (93–198)	126 (80–180)	0 (0–99)	100 (61–150)	136 (90–192)	130 (76–190)	130 (72–200)	130 (58–207)	131 (86–174)
	*n* = 388	159 ± 123	161 ± 121	149 ± 101	146 ± 80	150 ± 82	139 ± 80	58 ± 85	118 ± 96	147 ± 97	145 ± 115	144 ± 113	146 ± 117	139 ± 72

**^**a**^** = Significant mode group difference within a walking and cycling gender group, respectively; **^**b**^** = Significant gender difference within a mode group. The total commuting times per week in the dual mode groups have not been compared statistically with the total commuting time of the single-mode groups of each gender.

## 4. Discussion

This is the first study showing that male and female active commuting behaviors of walking and cycling in a metropolitan Nordic setting can be divided into three different modality groups (single-mode walking and cycling commuters, respectively, and dual-mode commuters) and that these groups have both important similarities and differences in their behaviors. For example, the median trip durations are rather stable for these groups and for gender (25–35 min per trip), when not considering the dual mode group when cycling, whereas the trip frequency varies greatly over the year depending on the modality. While the single-mode pedestrians’ median trip frequency is high and stable over the year, the cyclists’ median trip frequency is low or non-existent during the period of November–March. This overall cycling scheme refers to both gender and single-mode as well as dual-mode groups. However, the dual-mode commuters replace cycling with walking during the winter period. Note that, as indicated in Table 1, the temperature and precipitation patterns for the four indicator months during the year prior to when the respondents answered the questionnaire resembled the average values in the region for a longer period of time. Thus, both the seasonality in frequency of trips per week in different months and the high levels of the total number of active commuting trips over the year, ranging between 231 and 389 for the different modality groups, are most likely a close reflection of the normal pattern of active commuting in this region. 

The results regarding the extent to which the different modality groups and genders meet the minimal physical activity levels (150 minutes) per week with their active commuting behaviors indicate that this is the case for the single-mode pedestrians and that the dual-mode commuters are close to reaching these levels over the year, whereas this is the case during only six months of the year for the single-mode cyclists. Other results also indicate that, to a great extent, the commuting behaviors meet, or have the potential to meet, other temporal demands on physical activity for health enhancement, e.g., the minimum activity bout length of 10 min and 30 min of activity accumulated per day. A number of active commuters do also meet the optimal levels of 300 min per week during different periods of the year. Finally, the high numbers of trips per year add to the value of these behaviors from a public health perspective. These results will be discussed below.

### 4.1. The Metropolitan Setting As Study Area

From a public health point of view, an analysis of active commuting behaviors in a metropolitan setting is important since such urban living areas constitute one of the major human habitats globally. It is therefore clearly useful to understand the extent to which active commuting is feasible as a public health strategy in such areas. A crucial variable from this point of view is the distribution of trip distances between home and place of work or study within the population. Obviously, living and working in a small town constitutes a fundamentally different distance distribution than doing so within a metropolitan area, and this may also affect other variables, such as trip duration and frequency, and possibly velocity and the number of red-light stops.

We do not know the more precise commuting distance distribution within the studied region, but Statistics Sweden has divided Sweden into 82 functional labor markets areas, so-called LA areas, and Stockholm is the node of one of them. An LA area is defined as one or more municipalities in which no more than 20% of the workforce commutes to other municipalities. The functional labor market of Stockholm encompasses the whole of our study area and 10 neighboring municipalities with a total area of about 20,000 square km, *cf*. [16,17]. It is apparent that, for a certain proportion of the population, many commuting distances within such a wide area are too long to be covered solely by active commuting. Another consequence of such a study area is that the behavioral distribution of active commuting distances and durations is most likely to be substantially wider than in, for example, a small city. This means that within the higher levels of durations and distances covered by the active commuters in this study, we will probably find close to the maximal part of the distribution of values for this behavior in a population perspective. This is all the more plausible because there are good reasons to believe that our study group represents a more physically fit and motivated group than the corresponding general age groups in the population.

### 4.2. Duration, Distance and Velocity

The median values for the durations are remarkably stable when we consider the male and female single-mode pedestrians and cyclists, as well as dual-mode commuters when they walk (range, 25–35 min). In our mind, this is an indication of rather uniform reasoning about the issue of time budget allocation for active commuting between the modality and gender groups. Interestingly, Hu *et al.* [18] noted similar active commuting durations in the metropolitan area of Tianjin, China, with a substantially greater population (9 million) than Greater Stockholm. This may be taken as an indication that the noted behaviors in this study represent more or less maximal realistic behaviors with regard to durations in a metropolitan area.

The durations reported by the participants also include stops at red lights, and possibly also other types of stops. We do not know the exact amount of time these periods of inactivity account for; however, considering that we have a variability of between 1 and 3 red-light stops and, based on inspections of stop times in GPS tracings of active commuters in Stockholm, described in Stigell and Schantz [11], a reasonable value appears to be about 0.5–1.5 min. Thus, we see no reason why stops for red light would change the overall picture presented.

**Figure 5:**
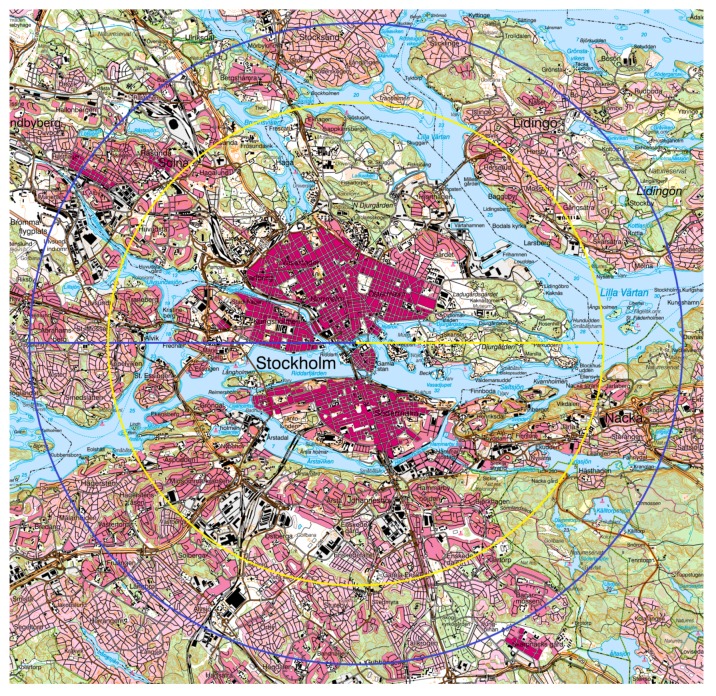
Map over the more central parts of the Greater Stockholm region, Sweden. Median distances for single-mode cyclists are indicated as the radius between the circles and the core of the inner urban area of Stockholm. The actual route distances (males 9.0 km, and females 6.7 km) have been transformed into the corresponding straight-line distances, which, on the average, are 80% of the actual route distances in this region [19]. The blue radius and circle represents males, and the yellow ones females. Within the yellow circle live about 350,000–400,000 inhabitants. Lantmäteriet^©^, Gävle, Sweden (I2014/00630).

As could be expected, the trip distances, as measured by a criterion method [15], vary between the modal groups. We observed about 15%–25% longer median distances for dual-mode than for single-mode pedestrians, and about 190%–290% longer distances were observed for the single-mode cyclists. This is, to our knowledge, the first time that such long commuting distances for single-mode commuting cycling (md, males, 9 km; females, 6.7 km), are reported, and it is reasonable to assume that this is an effect of the study area used, as well as the fact that we have separated single and dual-mode cyclists. We have converted these distances into their corresponding straight line distances [19] and illustrate them in Figure 5 as the radius in circles with the center placed in the core of the metropolitan area of Greater Stockholm. Within those circles we find both a high density of workplaces and a high residential density. For instance, within the yellow circle, about 350,000–400,000 of the region’s 1.9 million people live. Indeed, this is a quite typical European urban setting, and it points to the fact that a substantial portion of the inhabitants affected by this type of urban planning have realistic bicycling distances to their workplaces.

At the same time, given that our study was conducted in a widespread metropolitan area with a substantial proportion of distances that are too long for active commuting even by cycling, we believe that the distance data presented generally stand for behaviors in which the values above the median gradually approach the upper distance limits that can realistically be expected to be undertaken, at least within the segment of potentially new active commuters in the population. At this point of development in our understanding of active commuting behaviors, we suggest that the 3rd quartiles for trip distance in each modality group and gender can be used as a provisional indication of upper distance limits for active commuting behaviors within the population. Future studies should further our understanding of these issues through in-depth analyses of its dependence of age, work capacity, motivation, and bicycle gear number.

Another finding was that male single-mode cyclists cover longer distances than females. This is a reflection of higher cycling velocities among men, since the commuting time did not differ between the genders. There are several different possible explanations for this. Shorter all-mode commuting distances for women have been reported in Sweden [20,21,22], and for bicycle commuting in other countries [23,24]. Such differences in distance are often explained by the household responsibility hypothesis or by observations that female-dominated workplaces, like child and healthcare centers, have a more even spatial distribution in the urban area (*cf.* [25,26]). This could be interpreted in terms of women not having to cycle as fast as men to reach their workplaces within a certain commuting time allocation. However, since dual mode cyclists of both genders have about the same distance to work, and men still have higher cycling velocities than women, we believe that alternative explanations must also be searched for. For example, social norms acting via e.g., dress codes and acceptance of sweating, may influence the velocity of cycling, and this can also be due to higher demands on traffic safety among women [27,28]. Physiological explanations also need to be considered; whereas the leg muscle force capacity for walking and cycling appears to create rather similar conditions for both genders [29], the aerobic capacity relative to body mass has been shown to differ between genders (15%–20%) in a fashion that can, to a great extent, explain the differences in cycling velocity noted between men and women [30]. Finally, we note in this study that men generally have bicycles with a greater number of gears (Table 4). Whatever the reason is, the higher cycling velocities for men than for women in both the single and dual-mode groups are in accord with the results of other studies on bicycle commuting [31,32,33].

We also noted that dual-mode cyclists have lower velocities than single-mode cyclists (Table 5). An explanation for this might be the longer distances of single-mode commuting. In line with that, El-Geneidy *et al.* [34] have reported that longer distances in cycling as a mode of transport were associated with higher velocities. They reported that an increase in total distance of 1.6 km*, i.e.*, 1 mile, results in an 0.38 km/h higher velocity. A partial explanation of such a phenomenon could be that dual-mode cyclists use bicycles with a lower number of gears than single-mode cyclists (see Table 4). Another possibility is that the single-mode cyclists represent a subgroup with higher aerobic power than the dual-mode group, and thus that selection factors play a role here. Finally, the fact that the dual-mode cyclists, compared to the single-mode cyclists, cycle to a greater extent in the inner urban area (*cf.* Table 2 and Table 3) may also have affected their velocities, given that the inner urban and suburban areas constitute distinctly different traffic environments [10].

### 4.3. Seasonal Variation in Trip Frequency

To the best of our knowledge, this paper is the first to assess differences in self-reported walking and cycling frequencies between single and dual-mode active commuters of both genders. The reason for this is that single and dual-mode commuters are usually assessed jointly, and that commuters are categorized from their mode at the time of assessment [22]; thus, hiding a possible mode choice variability with the seasons. Furthermore, imprecise measuring methods have sometimes been used in reporting yearly frequencies. For example, activity diaries normally cover the last days’ or weeks’ travel, or population-based travel surveys and bicycle flow measurements that only encompass data or changes at an aggregated level. As a result, very little is known about how the behaviors of individual active commuters vary over the year.

Three striking characteristics are revealed in the present study. First of all, the high and stable trip frequency over the year noted among both male and female single-mode pedestrians. This stability is only interrupted during the month of July, and the cause for this is that July is the primary holiday month in Sweden. Second, this stability is in contrast with the high variability in cycling frequency for both genders among the single and dual-mode cyclists. Third, the variations in cycling frequency are replaced in the dual mode group of both genders by compensations in walking frequency. The latter finding underlines the importance of including questions about several active commuting modes, or at least walking and cycling, in trip and physical activity surveys; otherwise, the total active commuting frequency and physical activity levels might be underestimated.

Previous research has found seasonal variation in young adults’ total physical activity (see, e.g., [35]) and in leisure-time physical activity (see, e.g., [36]) with lower levels in the wintertime compared to other seasons. In line therewith, we note a rather dramatic seasonality in bicycle commuting among both single and dual-mode commuters. The decrease in cycle commuting between November and March is similar to the variations in flows of cyclists in the bicycle counts for arterial streets leading to the inner urban areas of Stockholm, as well as within the inner urban area [37,38]. It is also in line with findings from other cities in Sweden [39]. What is new in this study is that we note the same overall pattern of seasonality in both genders, and that it is seen to the same extent in single-mode cyclists with long distances as in dual mode cyclists with shorter distances. This is an indication that the cycling behaviors are very sensitive to changes in the external environment between the summer and winter halves of the year. This is strikingly different compared to the stability in the walking commuting behavior among single-mode pedestrians. To our knowledge, this has not been reported previously. McCormack *et al.* [40] assessed the seasonal variation in walking for transportation in Calgary in Canada. In contrast to the present study, they found a decrease in the wintertime. However, they assessed walking for transportation in general, and not walking to work specifically. As in the present study, they noted that the seasonal variation differs between different types of physical activity. For instance, they found significant seasonal differences in moderate-intensity physical activity, but not in vigorous activity.

What can explain the overall variations in cycling trip frequency compared to the stable frequency pattern of the pedestrians? When winter comes to Stockholm, many environmental changes occur in parallel: it gets darker, colder, and snow and ice may appear on the roads. It is therefore difficult to judge the effect of each individual factor. For instance, darkness could, hypothetically, affect the mode groups differently, but, at least in December and the adjacent months, the majority of the commuters in all three modal groups seem to start their commute before sunrise and return home after sunset (*cf.* descriptions of participants in Methods and Table 1). Therefore, this is not a plausible explanation for the differences between pedestrians and bicyclists.

Interestingly, the changeover in the external environment seems to affect men and women at somewhat different time points. Men continue to cycle for a longer period in the fall, and also to a greater extent during the winter, and those who stop cycling during the winter seem to start earlier in the spring than their female counterparts. Thus, there is a gender difference that stands out in a more detailed analysis of the seasonal variability. Possible reasons for this gender difference in the present study might be perceptions of more unsafe bicycling conditions and reduced comfort during the winter. Women appear to value the importance of personal and traffic safety higher than men (see, e.g., [34,35]) and they also appear to value cycling comfort higher than men (see [34]). Interestingly, for walking, we found no gender difference in commuting frequencies, so the explanation for the difference must include something specific for the cycling mode. Unlike walking, cycling includes keeping a vehicle in balance so as not to fall, and cyclists are therefore more exposed to poor road conditions. In line with this, Winters *et al.* [41] found that regular cyclists in Vancouver, Canada, perceived icy and snowy routes as major deterrents. However, the interaction with other traffic might also be a critical factor. The risk of not being seen by car drivers when cycling in darkness, and not seeing motorized vehicles, might be critical for the perception of safety. Finally, it should be mentioned that during the study period, and for several decades before it, managing the routes for cyclists in case of snowing has had a low priority. 

Interestingly, the median distance values for the dual-mode walkers are between 17% and 26% longer than in the single mode pedestrians. The number of walking trips in the dual mode group is, however, clearly lower than among the single-mode pedestrians, indicating that a walking commuting distance of about 3 km has passed the limit for many individuals as a high trip frequency behavior. In contrast, the long distances observed among single-mode bicyclists (males, 9 km; females, 6.7 km) are travelled with the same frequency during the summer half of the year as for the dual-mode cyclists, although the dual-mode commuters have 55%–68% shorter distances.

Future studies should assess walking and cycling with different purposes, as well as in combination with other transportation modes, from a perspective of seasonal variation, and should also explore the extent to which seasonal variations in active commuting can be overcome by means of improved winter route maintenance and better street-lighting. Indeed, to study these matters in different climate and daylight zones would be of clear value. 

### 4.4. Durations in Relation to Physical Activity Recommendations

There are several parts of the minimal recommended levels of physical activity of moderate intensity. One specifies that the physical activity bouts should be at least 10 min long. Another that one should accumulate 30 min of 10-minute or longer bouts, at least five days a week, thus amounting to a total of 150 min per week [5]. The latter figure agrees with the WHO physical activity recommendations from 2010 [6], which, however, include no statement about how many days one should spend to accumulate the 150 min. However, if more intensive exercise is undertaken, the time needed can be less [5,6].

With regard to the minimal recommendations, the first prerequisite of at least 10-minute-long physical activity bouts is met by more than 92% in most modality groups, but decreases to 75%–84% in the dual-mode group when cycling. Another prerequisite deal with the number of days per week. It is notable that a common peak frequency for the different modality groups is 8 per week. The most common pattern of commuting involves two trips per day (*cf.* Table 5), which would indicate that such days of active commuting are undertaken four days a week. Considering that about 90% of the individuals in most modality groups have 15-minute or longer commutes, this means that they accumulate at least 30 min of moderate physical activity per day during four days per week. This represents, as stated, the peak median behavior for most modal groups.

The third type of minimum physical activity demand relates to a total of 150 min of moderate activity. This is a figure based on 30 min five days a week [5]. Those recommendations do not discuss whether this volume of physical activity can be undertaken just as well on four days, but, as stated before, leave open the possibility of higher intensity physical activity on a lower number of days. Again, the WHO recommendations do not state anything about the number of days per week for the accumulation of 150 min per week [6]. However, in their interpretation statement, it is recommended that the physical activity is “spread throughout the week” and implemented as “regular physical activity throughout the week (such as five or more times per week)” [6]. No doubt, the median trip frequency patterns of all modality groups, fulfill these criteria when they implement their active commuting behaviors. The majority in the pedestrian group did also meet the 150 min part of the recommendation with exclusively commuting physical activity, except for in July, which is the predominant summer holiday month (Table 7, Figure 3 and Figure 4). The median levels of physical activity accumulated in the dual-mode group from cycling do not meet these guidelines. However, if walking and cycling times are added together, the dual-mode group has median values which, for most of the year, are only slightly lower than the physical activity recommendations (Table 7, Figure 3 and Figure 4). The median single-mode cyclists meet the recommendation solely in the spring–mid-fall part of the year (Table 7, Figure 3 and Figure 4). Thus, to meet the recommendations all-year round, the single-mode cyclists need to complement with other forms of physical activity in the wintertime. That is also true for the dual-mode group during most parts of the year, but to a rather modest extent. The dual-mode strategy therefore has clear advantages, and it is interesting to note that this seems to be a strategy that functions up to distances of about 4–5 km as judged from the 3rd distance quartiles of men and women.

The minimum recommendation is one thing; however, there are also recommendations saying that about 60 min of moderate-intensity physical activity per day signifies meeting more optimal levels of physical activity from a health-enhancing perspective [5,6]. Within most of the modality groups, about 50% have single-commute durations of 30 min or more (*cf.* Table 5). This means that if two commute trips per day are made, they will reach the optimal physical activity levels. On viewing both the number of trips per week and the total amount of physical activity time, it is apparent that a substantial portion of the active commuters accumulate optimal quantities of physical activity most working days of the week.

Duration represents, however, only one aspect of the physical activity recommendations, intensity being another. The calculated median velocities in the present study ranged from 5.2 to 5.4 km/h for walking and from 12.8 to 18.6 km/h for cycling. Are they intense enough to meet the recommendations? This is hard to tell, since they are based on valid distance values, but also on duration values that include time for red-light stops. Furthermore, due to rounding off in the majority of the cases, the duration values involve errors on the individual level, which, however, might cancel out at the group level. Nevertheless, with these limitations in mind, the median walking velocity in the present study is included in the proposed range of brisk walking in the recommendation of 3–4 mph or 4.8–6.4 km/h [42]. The lower limit in the recommendation corresponds to the first quartile of walking velocity in the present study, an indication that the velocity is in general brisk and of moderate intensity even if it also includes stops at red lights and junctions, *cf.* [43]. 

No velocity limits are set for cycling in the physical activity recommendation, but a moderate intensity correspond to 3–6 METs, and in the Compendium of Physical Activity, 4 METs correspond to 16 km/h [44]; hence, this is 1 MET higher than the minimal recommendation and an indication that the median bicycling velocities in the present study are of moderate intensity. Correspondingly, Haskell *et al.* [5] suggest that both walking and cycling, with a transportation purpose, should be recognized as being of moderate intensity. 

Finally, some comments follow here on the single-mode cycling strategy in relation to possible effects on health. If there is no substitution during the winter for the physical activity gained from cycling during the summer, a detraining effect will most likely take over rather quickly, leading to a lowered metabolic capacity as indicated by decreases in capillarization, levels of oxidative enzymes and the capacity for mitochondrial ATP production in skeletal muscle [45,46], and also a lowered glucose tolerance [47]. Interestingly, there is also a study on the effect of seasonal *vs.* all-year-round levels of physical activity on premature mortality, showing a beneficial effect only for the all-year-round physical activity [48]. Indeed, this is another and important reason to try to stimulate and enhance the conditions for all-year-round cycling.

### 4.5. Representativeness

In Stockholm, rather few people commute actively. In the most active period, *i.e.*, during the summer time, about 15%–20% of the work trips are by foot or bicycle the whole way [49] and, consequently, it is costly to randomly recruit an adequate number of active commuters to make between-group comparisons. This study sample was therefore recruited by advertisements. We believe that this recruitment method will, if at all, only moderately affect our study aims. This belief is based on several comparisons. In Stigell 2011 [50] the commuting behaviors of the advertisement-recruited sample were compared with those of the street-recruited sample of cyclists and a general concordance was noted. The only exception was in the yearly trip frequency, which was an expected difference between the samples since the street-recruited single-mode cyclists were gathered during the wintertime. Furthermore, to check if there was a clear selection bias, the sociodemographic characteristics of single-mode cyclists in our advertisement-recruited sample were compared with those of the street-recruited sample and a concordance was found. To further assess the representativeness of our results, we have compared our sample with a random population sample of active commuters from Greater Stockholm, recruited during the same period of time for the Regional Travel Survey 2004 (RVU04) [49]. The RVU04 was part of the evaluation of the Stockholm congestion charge trials and is described in detail elsewhere [51,52]. In brief, questionnaires and single-day travel diaries were sent to 77,000 individuals in Stockholm County in September and October 2004. About half of the individuals responded (*n* = 36,049) and of these, 1338 pedestrian and 990 bicycle commuters corresponded to our inclusion criteria. However, the RVU04 did not distinguish between dual and single-mode commuters; therefore, to be comparable with that sample, we aggregated single and dual modes in the present study to obtain integrated walking and bicycling groups (data not shown).

In the RVU04 the mean age was 41–42 years in the gender and mode groups, compared to 46–49 years in the present sample (see Table 2). There was a majority of female commuters in both the RVU04, 55%–59%, and in our sample, 59%–82% in the different mode groups. Furthermore, there were rather small differences in income, driver’s license possession and access to a car, with all samples having a higher income than the mean in the County and high access to a car and a high percentage with a driver’s license (data not shown). Overall, although there appear to be some differences between the three samples, on the whole, our advertisement-recruited sample seems to target a similar socio-economic group of active commuters to that of the RVU04 sample. Thus, taken altogether, the comparison between different samples suggests that socioeconomically speaking, the advertisement-recruited sample is reasonably representative of the population of active commuters in the study area.

### 4.6. Strengths and Limitations

The research was conducted in the metropolitan area of Stockholm, Sweden, where cycling and walking commuting are socially accepted behaviors. This strengthens the study in two ways. First it provides us with a more diverse study population in terms of more women and persons with a high socio-economic status than in other cultural settings where bicycling is regarded as the modal choice of low-income groups or fearless young men. Second, the Stockholm metropolitan area has a large “labor basin”, which gives a wide range of commuting distances, which, in turn, makes it possible to study active commuting behaviours when they are “stretched out” in terms of duration and distance. In addition, the sample is large enough to make reasonable between-group comparisons. It is also a strength that we make use of a criterion method for establishing the trip distances. 

However, there are also limitations to the study. Our participants were recruited by advertisements, which introduce a risk of selection bias. To decrease that risk, we stressed in the advertisement that we also welcomed commuters with very short commuting trips and the minimum number of trips per year was set to “at least once a year.” We also focused our analyses on between-group comparisons, which are presumably less affected by a potential selection bias. Other limitations are the use of a self-report questionnaire with an up to one-year recall of commuting frequencies. This might be a difficult task for the participants. However, since most of the participants are habitual commuters, this might reduce the complexity of the task since habits might be easier to recall than single episodes. 

The validity of the commuting time variable suffers from effects of the participants rounding off commuting times to multiples of five or ten, which is a phenomenon that seems to be common in surveys of travel behaviors [53]. If the commuting time is used to measure the duration of physical activity, this is a deficit. Furthermore, commuting is an intermittent activity with 1–3 stops per trip at red lights, and perhaps also at, e.g., busy intersections. It should also be mentioned that bicyclists might also stop pedaling downhill, or at high velocities. Thus, all commuting time is not physical activity time. We believe, however, that these pauses in physical activity have a minor influence on the commuting durations. The participants stated their mean commuting time at the time of receiving the questionnaire, but the duration might also vary in relation to seasons in a way that, in such a case, is not covered. Consequently, the trip distance might be a more robust measure of the active commuting “duration” than the stated commuting time.

## 5. Conclusions

Three different modal groups of both male and female active commuters have been identified in a Nordic metropolitan area. These modal groups should be considered in designs of future transportation surveys, studies, interventions, and promotion campaigns.

The modal groups are characterized by distinctly different active commuting behaviors in mainly trip frequency variation over the year, velocity, and commuting distance. Gender differences were noted regarding primarily distance and velocity in cycling. The commute distances noted among single-mode cyclists point to a great potential for cycling commuting within the population in urban settings.

There are various criteria within the recommendations for minimum levels of moderate physical activity for enhancing health. In view of the trip durations and the normal pattern of two commute trips per week, the commuting behaviors meet, to a very high extent, both the requirements of 10 min or more of physical activity bouts and of at least 30 min of physical activity per day of active commuting. A substantial portion of the commuters did indeed also reach optimal levels (60 min) of physical activity per day of active commuting. The median single-mode pedestrians of both genders meet the 150-minute per week recommendation for the major portion of the year exclusively by commuting, whereas the single-mode cyclists did so only during the spring–mid-fall. Thus, seasonal changes affect cycle commuting behaviors in a Nordic metropolitan setting. The median dual-mode commuters were close to meeting the 150-minute recommendation primarily due to their cycling in the summer, and supplemented with walking in the winter. 

Finally, also important is the finding of very high levels of active commuting trips over the year, ranging between 231–389 for the different modality groups. This facilitates sustaining the adaptive changes with physical activity and is thus an important argument for promoting active commuting for public health purposes. 
